# Distress Regulates Different Pathways in the Brain of Common Carp: A Preliminary Study

**DOI:** 10.3390/ani11020585

**Published:** 2021-02-23

**Authors:** Alexander Burren, Constanze Pietsch

**Affiliations:** School of Agricultural, Forest and Food Sciences (HAFL), Applied University Berne (BFH), 3052 Zollikofen, Switzerland; alexander.burren@bfh.ch

**Keywords:** aquaculture, stressors, carp, early immediate genes, biostatistics

## Abstract

**Simple Summary:**

The aquaculture sector provides for nearly half of the world’s seafood consumption, thanks to its large expansion over the last 30 years. Despite this intense growth, clear guidelines for responsible practices and animal wellbeing are lacking. Gene expression studies are a fundamental tool for understanding welfare, but stress-markers in aquaculture fish species are poorly studied. In addition, the biostatistical analysis of gene expression data is not trivial, and this study applies different statistical methods in order to evaluate potential differences in the gene expression levels between control fish and fish acutely stressed by exposure to air.

**Abstract:**

In this study, a stress trial was conducted with common carp, one of the most important species in aquaculture worldwide, to identify relevant gene regulation pathways in different areas of the brain. Acute distress due to exposure to air significantly activated the expression of the immediate early gene *c-fos* in the telencephalon. In addition, evidence for regulation of the two corticotropin-releasing factor (*crf*) genes in relation to their binding protein (corticotropin-releasing hormone-binding protein, *crh-bp*) is presented in this preliminary study. Inferences on the effects of due to exposure to air were obtained by using point estimation, which allows the prediction of a single value. This constitutes the best description to date of the previously generally unknown effects of stress in different brain regions in carp. Furthermore, principal component analyses were performed to reveal possible regulation patterns in the different regions of the fish brain. In conclusion, these preliminary studies on gene regulation in the carp brain that has been influenced by exposure to a stressor reveal that a number of genes may be successfully used as markers for exposure to unfavourable conditions.

## 1. Introduction

Distress is defined as a condition that interferes with the wellbeing of an animal if the adaptation processes of the animal fail to return the organism to physiological and/or psychological homeostasis [1,2]. Fish in aquaculture are often subjected to distress for short periods (acute stress), but can also be exposed to stressors for longer periods (chronic stress), meaning that the body’s biological functions are sufficiently altered and its coping mechanisms overwhelmed [1].

Understanding the immediate effects of stressors, such as handling and crowding, on gene regulation in fish, has been a focus of aquaculture research [3,4,5], in the interests of improving survival, growth, reproduction, and fillet quality. The brain, however, despite its role as first actor in the stress-cascade, has received comparatively little attention. Recently, a study on European seabass (*Dicentrarchus labrax* L.) and gilthead seabream (*Sparus aurata* L.) not only revealed relevant differences between species, but the importance of studying the different fish brain regions separately [6]. In addition, certain brain areas may even exhibit significant differences in gene expression [7]. The main purpose of this research is to identify stress-related biomarkers in different brain regions of common carp (*Cyprinus carpio*) to allow a precise evaluation of their rearing conditions.

The diversity of primary functions between the regions of a fish brain has been already studied in the past. The telencephalon, for instance, has long been regarded as solely fulfilling olfactory functions [8]. However, more recent research has confirmed its important role in the expression of emotional and motivational behaviour, as well as in fear conditioning in teleosts, including a pivotal role in these behaviours that are attributed to the amygdala [9,10,11]. The amygdala is known to play an essential role in mediating negative and positive emotions in mammals, which also involves the appraisal of incoming signals [12,13,14,15]. The optic tectum, which directly connects the efferent neurons with incoming retinal fibres in teleosts, was already recognized at an early stage as being essential for visually mediated behaviours already in early times [16,17]. However, the optic tectum is not solely required for the perception of motions, but has more recently been proven essential for the correct pacing of saccades during optokinetic responses in zebrafish (*Danio rerio*) [18]. In addition, the hypothalamus plays an important role in energy homeostasis and appetite regulation. The activated or suppressed neurons then lead to adjustments in behaviour and metabolism. Proopiomelanocortin (pomc) neurons appear to drive satiety in the hypothalamic arcuate nucleus of mice [19]. The cerebellum of fish is responsible for the coordination of body movements [20], and has been linked to spatial navigation [21].

The effects of stressors on the brain can be assessed by analysing the activities of different gene sets. Firstly, brain activity can be assessed, for instance, by measuring the increased expression of stress-related immediate early genes (IEGs). One example of an IEG is *c-fos*, which, due to its very rapid and robust expression, is widely used as a functional marker of neuronal activity after a diversity of stimuli in vertebrates [22]. In fish, different stimuli have been shown to induce varying levels of *c-fos* expression. Light avoidance, as an innate choice behaviour, involves rapid changes in the expression of *c-fos* in the medial zone of the dorsal telencephalon in adult zebrafish [11]. In addition, the administration of D-amphetamine, known to activate the reward system, resulted in increased expression of *c-fos* in the same brain region 30 min after the injection of the substance [9]. Moreover, the sleep and wake behaviour of zebrafish leads to typical differences in *c-fos* patterns in zebrafish [23]. Even caffeine has been proven to act as a stimulator of *c-fos* in the zebrafish brain [24]. In addition, exposure to neurotoxins for 60 min resulted in rapid changes of the c-fos protein in different regions of the brain in killifish (*Fundulus heteroclitus*) [25].

Another gene belonging to the IEG group is *egr-1* (encodes the early growth response protein 1), which has already been shown to undergo change during the breeding cycle of sticklebacks [26]. Furthermore, the immunohistochemical detection of phosphorylated extracellular signal–regulated kinase (erk) has also been used as a read-out for neural activity in fish at the whole-brain level [27]. The protein palladin (palld) is essential for the organization of the actin cytoskeleton and a deficiency can lead to a failure of neurite outgrowth in rats [28]. Interestingly, palld has also been described as an immune-related gene in mirror carp (*Cyprinus carpio*) exposed to koi herpes virus [29]. The importance of this protein in cytoskeleton organization and kidney function has been confirmed in zebrafish [30,31]. Since it may also play an important role in the organization of the fish brain, this gene was included in this study. In addition, one metabolic gene (glyceraldehyde-3-phosphate dehydrogenase, *gadph*) was included in this study as its activity in tissues implicates higher energy demands and, therefore, increases in metabolic gene expression.

Secondly, the response to stressors also commonly involves activation of the hypothalamus-pituitary-interrenal (HPI) axis genes [32] and a number of these genes have therefore also been included in study. The corticotropin-releasing factor (crf) and its receptors play an important role in stress signalling via the HPI axis [33]. The abundance of the corticotropin-releasing hormone-binding protein (crh-bp) determines the availability of *crh* to its receptors, although other biological functions have been proposed for crh-bp [34]. Finally, the release of stress hormones, such as cortisol and 11-deoxycorticosterone, leads to the activation of glucocorticoid receptor (gr)- and mineralocorticoid receptor (mr)-mediated signalling pathways in teleosts [35]. In carp, gr2 is the most sensitive corticoid receptor, followed by mr, and gr1a and gr1b [36].

Thirdly, other brain networks are also known to be involved and/or affected by stress responses in other fish species. One example involves the serotonergic pathways being affected by stress in rainbow trout, *Oncorhynchus mykiss* [37]. Serotonin also plays a role in the habituation to startling acoustic stimuli in zebrafish [38]. While serotonin agonists have anxiolytic effects in humans [39], chemicals including ethanol have shown that the acute anxiolytic effects caused by these substances are likely to be mediated by γ-aminobutyric acid receptors A (gabaa, [40]). The same psychoactive compounds have also been used to influence normal behaviour in zebrafish [41]. In addition, together with vasotocin, isotocin is a neurotransmitter and neuromodulator that is produced in distinct neurosecretory neurons in the hypothalamic nuclei [42]. Both influence the outcomes of various behaviours, the establishment of social status, but are also involved in osmoregulation and stress responses in fish [42]. Osmoregulation is also regulated by prolactin in fish [43]. In addition, early development, behaviour, growth, and immunoregulation depend on prolactin and prolactin receptor expression [30,44]. In neuronal tissue, prolactin is also involved in the activation of neurons that evoke action potentials and/or calcium influx in neurons [45]. These reactions culminate in the release of neurotransmitters, e.g., dopamine in the hypothalamus of rats, which exert a negative feedback on the release of prolactin [46,47,48,49]. Interestingly, GABA-mediated inhibition of prolactin release from the pituitary of trout, probably by both, GABA receptors A and B, has been described by Prunet et al. [50]. However, the influence of stressors on these brain regulation pathways in other fish species is mostly unknown, which is the reason why a wider range of genes was included in this study.

This study was conducted to obtain preliminary data on the differential gene expression patterns of genes belonging (I) to the early immediate genes, (II) to the HPI axis, as well as (III) neurotransmitter-related pathways in the carp brain of stressed fish compared with non-stressed animals, used as controls, and to apply different biostatistical methods that allow the identification of a set of potential genes suitable as biomarkers of distress in fish.

## 2. Materials and Methods

### 2.1. Rearing Conditions and Sampling

The fish were reared at 23 °C–24 °C in a 290 L aquarium equipped with a settler and a moving-bed biofilter. The juvenile carp (*Cyprinus carpio*), approximately 2-months old, were kept for two months and fed 4 times daily at a feeding rate of 2 to 3% body weight per day. During the experiment, the mean weight of the eight fish was 28.3 ± 9.8 g and the mean standard length was 8.9 ± 1.1 cm. For stress treatment, four fish were exposed to the air for 1 min in a net, returned to the tank and anaesthetized 30 min afterwards. Following the acclimatization period, the four control fish were taken directly from the rearing tank. Anaesthesia was performed with an overdose of tricaine methanesulfonate (MS-222, Sigma-Aldrich, Buchs, Switzerland). The brains were sampled and stored in RNA*later*^®^ (Sigma-Aldrich, Buchs, Switzerland) for at least 24 h and then divided into the 4 brain regions (tel = telencephalon, hyp = hypothalamus, opt = optic tectum, rho = rhombencephalon, comprising the corpus cerebelli and the medulla oblongata). All experimental procedures were approved under permission number ZH-062-17 by the Cantonal veterinary authorities of Zurich (Switzerland).

### 2.2. PCR Conditions

Gene expression studies were performed by means of quantitative real-time polymerase chain reactions (qPCR) on a LC480 Light Cycler II (Roche, Basel, Switzerland). The total RNA from each of the four regions of each brain, tel, hyp, opt, and rho, was extracted using RNeasy Micro Kits (Qiagen AG, Hombrechtikon, Switzerland). The RNA content was confirmed using a spectrophotometer Q5000 (Quawell, San Jose, CA, USA). Subsequently, 20 μL of total RNA were reverse transcribed into cDNA using a High-Capacity cDNA Reverse Transcription Kit (Applied Biosystems, distributed by Thermo Fisher Scientific, Basel, Switzerland) according to the manufacturer’s instructions. Thereafter, the cDNA content was adjusted to 50 ng per μL using nuclease-free water (Ambion^®^, distributed by Thermo Fisher Scientific, Basel, Switzerland) and used for real-time PCR with the LightCycler^®^ SYBR^®^ Green I Master mix (Roche, Switzerland) using three replicates for each sample with the following cycling conditions: initial denaturation at 95 °C for 15 min, followed by 40 cycles at 95 °C for 15 s, 60 °C for 30 s, and 72 °C for 30 s. A melt curve step (95 °C for 5 s, 60 °C for 1 min, and 5 acquisitions per °C until 97 °C was included using a ramp rate of 0.06 °C per s) was added at the end of all runs to ensure that the individual PCR reactions yielded a single melting peak. All primer pairs used here are given in Table S1. Initially, the PCR products were mixed with the tracking dye orange G ((Thermo Fisher Scientific, Allschwil, Switzerland) and loaded onto 2% agarose (Bio-Rad Laboratories AG, Cressier, Switzerland) gels containing GelRed^®^ (Chemie Brunschwig AG, Basel, Switzerland) and after horizontal gel electrophoresis in TAE buffer (242 g TRIS-HCl, 37.2 g sodium EDTA, 57.1 mL glacial acetic acid in 1 L distilled water, pH 7.5) for 35 min at 80 mV PCR products were visualized using UV illumination. In addition, for primer validation, the respective PCR products (with sizes ranging between 90 and 150 bp) confirmed by Sanger sequencing and the optimal reference genes were extracted from a set of 8 possible reference genes (Table S2). This was accomplished using the three genes with the best value for the expression stability M (i.e., *eIF4E*, *bactin*, and *ef*, for more details see Table S2), extracted with the geNorm function in the qbase+ software, version 3.0 (Biogazelle, Zwijnaarde, Belgium—www.qbaseplus.com accessed on 5 March 2020) established by Vandesompele et al. [51]. The target genes included early immediate genes (*c-fos*, *egr-1*, *erk-1*, *erk-2*, and *palld*), as well as the metabolic gene *gapdh*, in order to indicate active brain regions. The following genes related to the HPI axis were included: *crf1*, *crf2*, *crfr1*, *crfr2*, *crhbp*, *pomc1*, *pomc2*, *gr1*, *gr2*, and *mr*. In addition, serotonergic pathway genes (*5-ht-r*, *serotr*) as well as *gabaa, iso-pre* and *prolr* were investigated.

### 2.3. Calculations and Statistics

From the potential reference genes *18S RNA*, *b_2_m*, *egr-1*, *palld*, *gapdh*, *bactin*, *eIF4E*, and *ef*, the reference genes that were used for gene normalization were *bactin*, *eIF4E*, and *ef* since these genes showed the lowest M value as calculated by the qbase+ software (Biogazelle, Zwijnaarde, Belgium, Table S2). The calculation of the normalized fold change in expression of each target gene was calculated according to Taylor et al. [52]. First, the mean quantitative cycle (mean ct) of the three technical replicates was calculated for each sample. The average ct of all control samples was calculated for each target gene and the relative difference (Δct) between the average ct for the control group and the mean ct for each sample was assessed within each target. Relative quantities are then calculated from the Δct values. For each biological group (i.e., control group versus air-exposed group), a normalization factor was derived from the geometric mean of the relative quantities of each reference gene. The relative quantity of each target gene was then divided by the normalization factor, followed by log transformation. The values hereby obtained were then used to calculate the geometric means for each treatment group. The standard deviation (SD) and the standard error of the mean (SEM) were calculated from the log transformed normalized expression data. The figures show the average relative normalized expression for each target gene ± SEM. Non-parametric tests (Mann–Whitney U tests) were run in IBM SPSS Statistics (version 26, IBM Schweiz, Zurich, Switzerland) to investigate the differences between the means for gene expression in each treatment group, since it has previously been shown that non-parametric methods may be better at controlling for false discoveries of significant differences between expression levels, for example, after RNA sequencing [53]. Differences between treatment groups were considered statistically significant when *p* < 0.05. Confidence intervals (95%) for the values of control and stressed fish have been derived by resampling 2000 times from the original sample based on the adjusted bootstrap percentile (BCa) method using the *resample* and the *broom* package in R studio. Assuming the independence of tests, multiple testing leads to an inflated probability of false positive results. To address this problem, the following mixed models with a fully Bayesian approach (as a part of the *brms* package [54] in R studio, Version 1.2.1335, RStudio Team 2018) and assuming a Gaussian distribution of the data were used to investigate potential differences between the two treatment groups:

y_ij_~N(μ_ij_, s^2^) (1)

μ_ij_, ~α_j_ + β_j_*x*_i_ + *y*_i_ (2)

α_j_~N(0, σ _α_^2^), 1, …, n_gen_ (3)

β_j_~N(0, σ _β_^2^) (4)

*y*_j_~N(0, σ *_y_*^2^), *i* = 1, …, n_animal_ (5)

The models include gene specific random effects for the constants (α), gene-specific random effects for the group differences (β) and animal-specific random effects for the constants (γ). The model fit was assessed through a comparison of graphical plots (QQ plots) showing the distribution of y and y_rep_. To improve the handling of possible outliers, posterior predictive checks based on the Markov Chain Monte Carlo (MCMC) approximation method were applied, which yielded simulated replicated data under the fitted model that were then compared with the observed data. The point estimators, their SEMs, credibility intervals and posterior predictive *p* values are reported.

A principal component analysis (PCA), as a data reduction method, was performed on the log transformed normalized expression data in R studio (Version 1.2.1335, RStudio Team 2018) for an initial description of the genes accounting for the main contribution towards the common variance within the gene expression patterns in the different brain regions. The representation of the variables for the principle components is calculated as a cos^2^ value. For a given variable, the sum of the cos^2^ on all the principal components is equal to one.

## 3. Results

### 3.1. Immediate Early Genes (c-fos, egr-1, erk-1 and erk-2, palld) and gapdh

**Figure 1:**
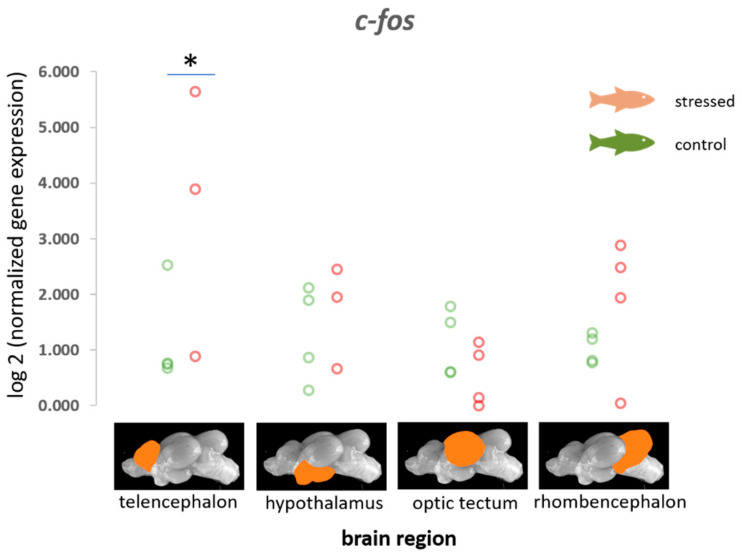
Gene expression profile of the immediate early gene *c-fos* in each of the four brain parts in the control fish and fish 30 min after the air exposure with 95% confidence intervals of 0.72 to 2.1 for the control and 2.1 to 18.5 for the stressed fish for the values for the telencephalon derived from the adjusted bootstrap percentile (BCa) method; *n* = 4 per treatment, * = significance according to the Man–Whitney U tests, *p* < 0.05.

A significant difference was observed between control fish and distressed fish for the IEG *c-fos* in the telencephalon (*p* < 0.05), but not in the other brain regions that were investigated (Figure 1). In addition, an increased probability for a reduction in the expression of this gene was observed in the optic tectum relative to other genes that were included in this study (Table 1 and Figure S4). However, the other IEGs were not significantly influenced by the stress treatment.

For the IEGs, the first two components in the PCA for each brain region explained 77.1% of the variance in the IEG data in the telencephalon, 83.8% in the hypothalamus, 82.8% in the optic tectum, and 81.1% of the variance in the rhombencephalon (Figure S1).

However, the genes that indicated good representation on the principal component, i.e., displayed by a high cos^2^ value in Figure S1, differed for each brain region.

### 3.2. HPI Axis-Related Genes

**Figure 2:**
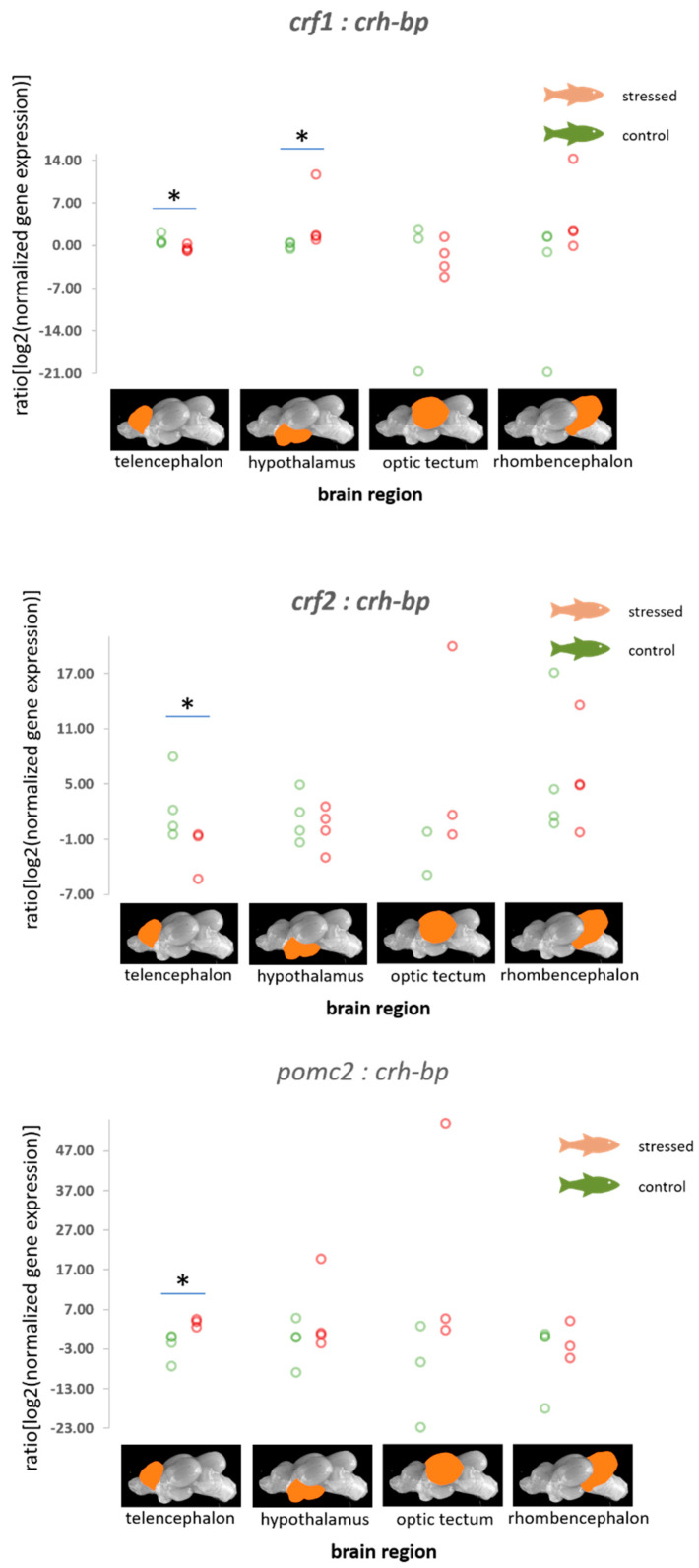
Ratios of the normalized fold expression of *crf1, crf2*, and *pomc2* relative to *crh-bp* in each of the 4 brain parts in the control fish and fish 30 min after the air exposure with 95% confidence intervals of 0.51 to 1.83 for the control and −0.77 to 0.17 for the stressed fish for the ratio of *crf1:crh-bp* in the telencephalon, −0.44 to 0.53 for the control and 1.16 to 9.20 for the stressed fish for the ratio of *crf1:crh-bp* in the hypothalamus; 0.00 to 6.48 for the control and −8.07 to −0.61 for the stressed fish for the ratio of *crf2:crh-bp* in the telencephalon, and −7.16 to −0.13 for the control and 2.62 to 4.42 for the stressed fish for the ratio of *pomc2:crh-bp* in the telencephalon derived from the adjusted bootstrap percentile (BCa) method; *n* = 4 per treatment, * = significance according to the Mann–Whitney U tests, *p* < 0.05.

For stress responses, it is essential to consider how the mRNA expression values of hormones, their binding proteins and receptors change relative to each other. A significant decrease in the ratio of *crf1:crh-bp* in the telencephalon and an increase in the hypothalamus were observed in stressed fish compared with the control fish (*p* = 0.021, Figure 2). Furthermore, the ratio of *crf2:crh-bp* was decreased in the telencephalon though the application of stress (*p* = 0.029, Figure 2).

**Figure 3:**
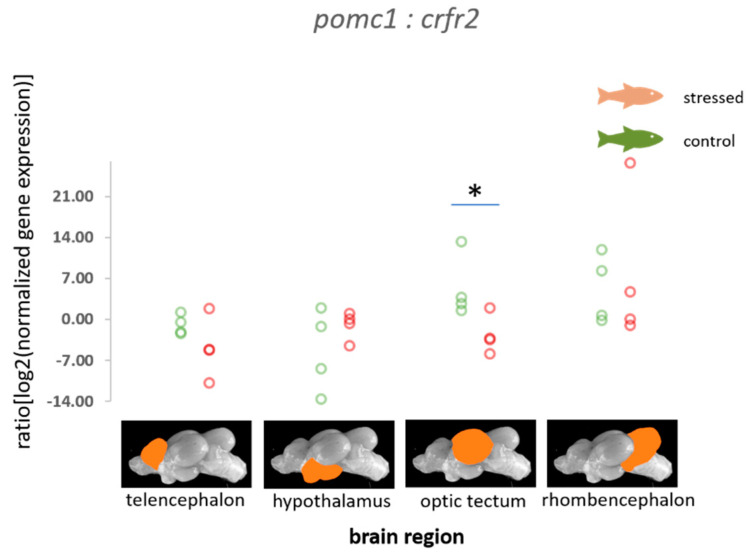
Ratios of the normalized fold expression of *pomc1* compared with the expression of *crfr2* in each of the four brain parts in the control fish and fish 30 min after the air exposure with 95% confidence intervals of 2.14 to 10.98 for the control and −5.17 to 0.63 for the stressed fish for values for the optic tectum derived from the adjusted bootstrap percentile (BCa) method; *n* = 4 per treatment, * = significance according to the Mann–Whitney U tests, *p* < 0.05.

In addition, the ratios of the normalized fold change in expression of *pomc2* relative to *crh-bp* were found to be increased in the telencephalon of stressed fish compared with the control fish (*p* = 0.034, Figure 2). The ratio of the normalized fold change in expression of *pomc1* to the *crf* receptor 2 was found to be significantly decreased (*p* = 0.043, Figure 3).

The PCA revealed that the first two components of the PCA represented 74.5% of the total variance in the HPI genes in the telencephalon, 74.2% of the variance in the same genes in the hypothalamus, and 78.8% and 60.6% of the variance in these genes in the optic tectum and the rhombencephalon (Figure S2). A high cos^2^ value was observed for *crf-r2* in all four brain regions, which indicates good representation of this variable on the principal component. In these cases, the variables are positioned close to the circumference of the correlation circle in Figure S2. In contrast, a low cos^2^ indicates that the variable is not perfectly represented by the principal components. In this case, the variable is close to the centre of the circle, which, for example, can be seen for *pomc2* in the telencephalon and rhombencephalon (Figure S2).

### 3.3. The Gene Expression Patterns of the Serotonergic Genes, gabaa, Isotocin Precursor, and the Prolactin Receptor

**Figure 4:**
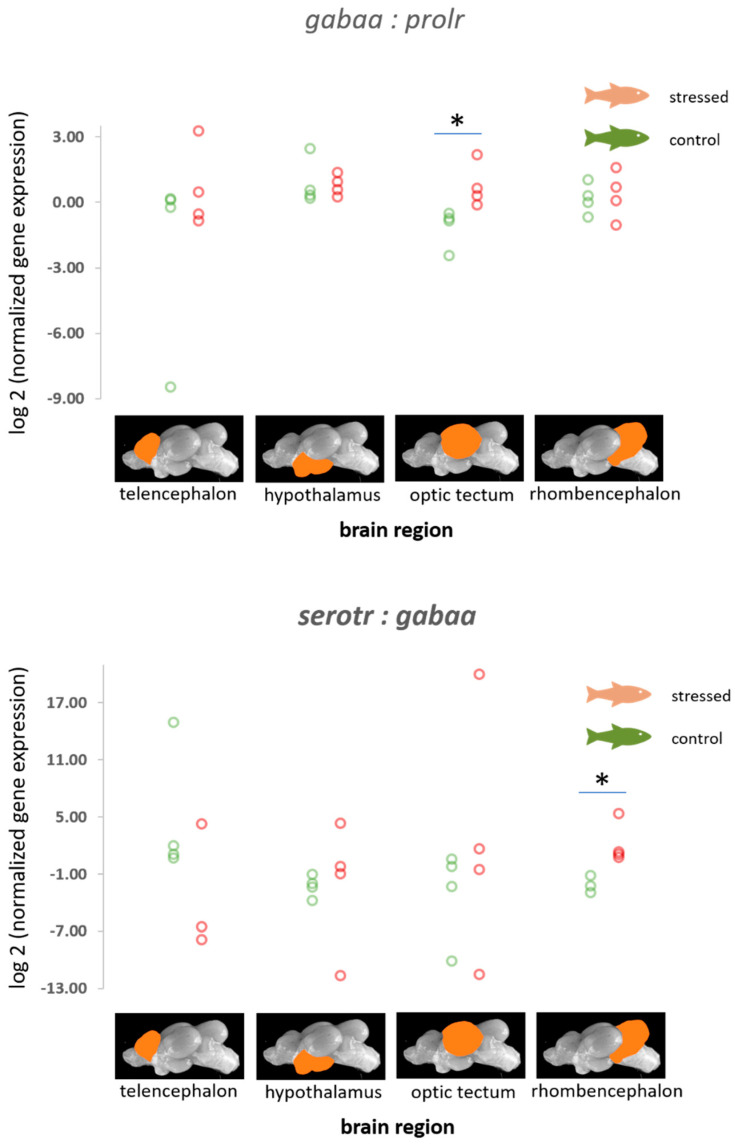
Ratios of the normalized fold expression of *gabaa* to *prolr* and the normalized fold expression of *serotr* to the expression of the *gabaa* gene in each of the four brain parts in the control fish and fish 30 min after the air exposure with 95% confidence intervals of −2.43 to −0.63 for the control and 0.105 to 1.813 for the stressed fish for the *gabaa:prolr* ratio in the optic tectum, and −2.92 to −1.49 for the control and 0.95 to 4.39 for the stressed fish for the *serotr:gabaa* ratio in the rhombencephalon derived from the adjusted bootstrap percentile (BCa) method; *n* = 4 per treatment, * = significance according to the Mann–Whitney U tests, *p* < 0.05.

The ratios of normalized fold change in expression of *gabaa* relative to *prolr* were found to be increased in the optic tectum of stressed fish compared with the control fish (*p* = 0.021, Figure 4). Furthermore, calculations revealed an increased probability of a reduction in *gabaa* expression in the optic tectum relative to other genes in this study (Table 1 and Figure S4). In addition, the ratio of normalized fold change in expression of *serotr* relative to *gabaa* was found to be increased in the rhombencephalon of stressed fish compared with the control fish (*p* = 0.021, Figure 4), as well as an increased probability of a reduction in *serotr* in the telencephalon relative to other genes that were included in this study (Table 1 and Figure S4).

**Figure 5:**
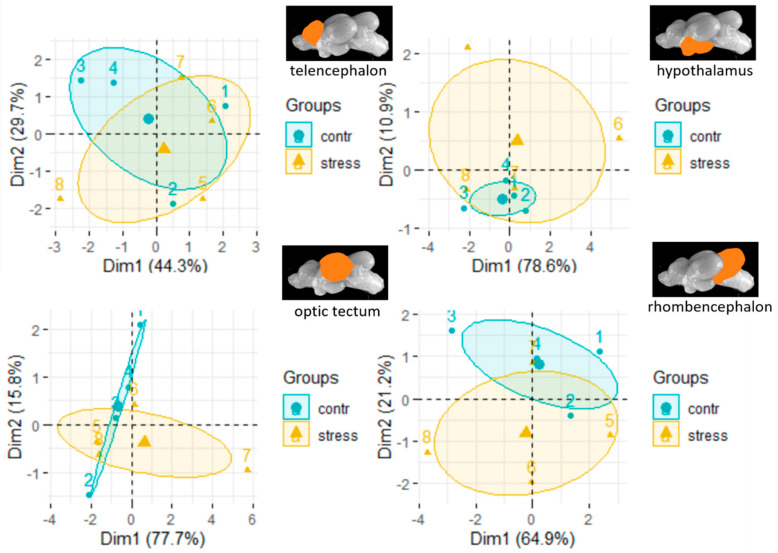
Results for the first two components (Dim1 and Dim2) of the principal component analysis (PCA), including confidence ellipses for the selected genes *gabaa*, *crfr1*, *crfr2*, *erg-1*, *mr*, *erg-1*, *5-ht-r* and *c-fos* in each in the four brain parts of control fish and fish 30 min after the air exposure (the numbers in the brackets indicate the percentage of the variance in the data sets that is explained by each components, mean ± SEM; *n* = 4 per treatment.

The PCA for the mRNA levels of the genes *5-ht-r, serotr, gabaa, isopre*, and *prolr* in the telencephalon explained 72.2% of the variance in the data set, whereas the same calculations in the hypothalamus revealed that 85.9% of the variance is related to the selected genes (Figure S3). Similarly, 83.6% and 73.9% of the variance was attributed to the selected genes in the optic tectum and in the rhombencephalon, respectively (Figure S3). Consequently, an optimal set of genes was desired for a final PCA, for which *gabaa*, *crfr1*, *crfr2*, *mr*, *egr-1*, *5-ht-r*, and *c-fos* were selected for each of the individual brain regions (Figure 5). The PCA for the telencephalon revealed that 74.0% of the variance in the data set was explained when these genes were selected as variables. Compared to this, the PCA for the hypothalamus showed that 89.5% of the variance was covered by the selected genes. Similarly, the respective variance levels were as high as 93.5% in the optic tectum and 86.1% in the rhombencephalon.

## 4. Discussion

Fish reared in aquaculture systems are continuously exposed to different stimuli, some of which are capable of inducing a distress situation once they overcome the physiological limits of the animals. With the continuous growth of the fish farming industry, ways to define and quantify welfare are becoming vital to producing recommendations for best practice and adaptations to legislation. In this study, we aimed to identify the diversity of gene expression profiles between the four brain regions (telencephalon, optic tectum, hypothalamus, and rhombencephalon) in distressed common carp.

Reference genes are fundamental to the exact determination of changes in gene expression [52,55]. These are usually chosen based on previous studies, but only a limited number of these have selected reference genes according to their stable expression pattern in distinct tissues [56,57]. Typical reference genes are related to maintenance of cell structures and metabolism. No such detailed investigation of suitable reference genes has been performed before for the fish brain. The reason for this is that even acute stress, for example in trout, can alter the gene expression of a number of genes involved in intracellular signalling and cytoskeletal changes [58]. Thus, the selection of housekeeping genes that typically have these functions in cells might be inadequate. Other genes, such as *gapdh*, may exhibit a high variability, which makes them unsuitable as references genes [59,60]. Here, only three genes were shown to be suitable as reference genes. Given the limited number of animals per treatment group, and in order to confirm the hypothesis that region-specific reference genes are required for the fish brain, an additional study using a higher sample size is recommended to confirm the suitability of the reference genes analysed in brain regulation studies on fish.

### 4.1. Immediate Early Genes (IEGs)

Different IEGs were investigated in this study to identify brain activity in the various brain regions. The increased expression of *c-fos* in the telencephalon of distressed carp and the increased probability of a reduction in the expression of this gene in the optic tectum relative to other genes (cf. Figure S4) confirms that *c-fos* is not only a suitable indicator of brain activity in higher vertebrates and fish species, such as zebrafish and goldfish [11,48,61], but also in carp. In situ hybridization has already shown that light avoidance leads to changes in the expression of *c-fos* in the medial zone of the dorsal telencephalon in adult zebrafish 30 min after the induction of neuronal activity [11]. Together with the current results from carp, it becomes clear that *c-fos* is capable of indicating changes in brain activity after exposure to acute stressors, in this case exposure to air.

### 4.2. HPI Axis-Related Genes

The fact that acute stress involves the crf system, e.g., the pre-optic area in the forebrain in fish, has already been reviewed in the past [61,62]. The investigations of acute and chronic stress responses and the crf system also included farmed fish species, such as rainbow trout, sea bass and gilthead seabream, but not carp [6,33,63]. The wide distribution of crf and crh-bp in the fish brain supports the assumption that the crf system fulfils important functions, even outside the cerebral system [64]. For example, changes in the crf system caused by stressors, including hypoxia, have also been observed in the caudal neurosecretory system and the heart of zebrafish [65,66]. Crh-bp is known to inhibit the crf-mediated activation of the crf receptors in a receptor subtype-specific fashion [67]. For this reason, the ratios of the normalized fold change in expression of the *crf* genes in this study were compared to the level of *crh-bp*, and the application of an acute stressor appears to influence this ratio, proving the assumption that the crh-bp actions are receptor-specific. Previous investigations have indicated that crh-bp is a more potent inhibitor of crfr2 activation than of crfr1 in fish [67]. Similarly, the crf2 receptors in humans have been described as being coupled to the cAMP–PKA signalling pathways similar to crfr2, but they mediate effects opposite to those of crfr1 receptors in arthritis [68]. This reflects the fact that crf1 and crf2, as well as the two crf receptors have different functions, which is probably also the case in fish. The differential effects of distress on the ratio of the normalized fold change in expression of the *crf* receptors compared with the fold change in expression of *crh-bp* in this study support the assumption that the two receptors have different functions in carp as well.

In this study, the ratio of the normalized fold change in expression of *pomc1* to *crfr2* is also reduced in the optic tectum. The hypothalamic circuit includes two populations of neurons: one co-expressing orexigenic neuropeptides, such as neuropeptide Y, and the other expressing pro-opiomelanocortin (*pomc*) and anorexigenic neuropeptides, thus regulating feed intake in fish [69]. The functions of pomc neurons in the optic tectum are less well described. Nevertheless, the results of this study support the hypothesis that the optic tectum is more than a predominantly retinorecipient structure.

### 4.3. Genes of the Serotonergic and the GABAergic Pathway

A link between prolactin release and GABA receptor signalling has previously been demonstrated in the hypothalamus of rodents, as well as in the pituitary in rodents and rainbow trout [47,49,50]. Surprisingly, the change in the ratio of the normalized fold change in expression of the GABA A receptor (*gabaa*) relative to the expression of *prolr* was observed in the optic tectum, but the role of *prolr* in this region of the brain remains unclear to date. The levels of GABA receptor mRNA expression, together with the expression of other important receptors, such as membrane receptors for serotonin and dopamine, affect memory loss in rats [70].

Serotonin transporter expression and activity is required for returning serotonin to the presynaptic neuron where it can be degraded or retained for later re-use. In higher vertebrates, selective serotonin reuptake inhibitors may lead to increased GABA concentrations [71], confirming an interaction between serotonin pathways and GABA levels. The current study on carp also indicated that the ratio of *serotr* to *gabaa* is influenced by acute distress. In trout, acute stress resulted in downregulation of a serotonin receptor subtype and *mr* in the telencephalon 4 h post-stress when compared with the levels 1 h post-stress, which indicates that a negative feedback exists for these receptors, that aims to downregulate the HPI axis after activation by stress [72]. More sampling time points would have been required in this study to show the dynamics of the activation of similar feedback mechanisms in carp.

In rodents, maternal care increases the 5-ht turnover at the serotonin receptor, elevating the activity of this receptor, which leads to the activation of expression of factors, such as *egr-1* further downstream [73,74]. The PCA in the present study indicated that *egr-1* is a suitable gene for indicating changes in brain regulation in carp as a result of exposure to stress.

## 5. Conclusions

Acute distress by air exposure regulates gene expression differently in the brain regions of common carp. This study, therefore, performed an analysis of gene expression stability of potential reference genes before calculation the changes of mRNA expression changes of target genes. The PCA showed that a relatively low number of variables (i.e., genes for which their mRNA expression was investigated) is sufficient to explain a high amount of variance within the data set. In the optic tectum, this amount of variance was found to be higher than 90%. This is a promising result to better identify optimal genes as markers for stress in fish and confirms that, besides the known HPI axis gene, genes belonging to the group of immediate early genes and to neurotransmitter pathways should also be included in future biomarker development studies. However, the basic concept of a PCA is that the components contribute to the common variance, which is certainly not an optimal assumption for gene expression data in the brain. The regulation of genes in the brain, especially as a result of a (dis-)stressor, probably leads to a common regulation pattern, as well as a unique regulation of certain genes. Therefore, exploratory factor analysis is currently assumed to yield better models for the description of gene regulations in the fish brain. Given the limited number of samples analysed in the present study, the calculations are preliminary ones. In the future, a broader study with a higher number of animals per treatment group and additional stressors will be conducted and will highlight more detailed insights in the gene regulation in carp. Furthermore, studies considering the genetical background of farmed fish would be needed to show the relevance of stress biomarkers under farm condition. In addition, future research on brain activity will have to include a number of physiological and behavioural parameters allowing the more precise assessment of stress responses in fish.

## Figures and Tables

**Table 1:** Probabilities for potential group differences between the control animals and the distressed fish (*n* = 4 each) in the different brain parts, the table shows for each of the genes the point estimator displayed in bold, the SEM in brackets, and the credibility interval and the posteriori *p* value in the second row.

Gene	Tel *	Hyp **	Opt ***	Rho ****
*18S RNA*	**0.16 (0.59)** −1–1.39, *p* = 0.602	**0.29 (0.46)** −0.5–1.31, *p* = 0.740	**0.75 (0.83)** −0.76–2.47, *p* = 0.824	**0.35 (0.46)** −0.52–1.28, *p* = 0.780
*5-ht-r*	**0.22 (0.60)** −0.9–1.51, *p* = 0.646	**0 (0.41)** −0.87–0.86, *p* = 0.492	**0.09 (0.82)** 1.57–1.77, *p* = 0.540	**0.03 (0.45)** −0.87–0.91, *p* = 0.526
*eIF4E*	**−0.01 (0.60)** −1.22–1.22, *p* = 0.496	**−0.48 (0.51)** −1.66–0.31, *p* = 0.162	**−0.66 (0.85)** −2.41–0.98, *p* = 0.210	**0.03 (0.46)** −0.90–0.96, *p* = 0.520
*b_2_m*	**0.01 (0.58)** −1.15–1.18, *p* = 0.515	**0.09 (0.42)** −0.75–1.01, *p* = 0.580	**−0.85 (0.82)** −2.58–0.64, *p* = 0.144	**0.15 (0.44)** −0.72–1.07, *p* = 0.630
*bactin*	**0.13 (0.60)** −1.06–1.38, *p* = 0.584	**0.14 (0.43)** −0.67–1.12, *p* = 0.621	**0.33 (0.82)** −1.23–2.03, *p* = 0.650	**−0.08 (0.45)** −1.00–0.80, *p* = 0.432
*c-fos*	**0.91 (0.70)** −0.26–2.40, *p* = 0.912	**0.37 (0.48)** −0.40–1.49, *p* = 0.783	**−1.5 (0.93)** −3.41–0.21, *p* = 0.045	**0.33 (0.46)** −0.53–1.28, *p* = 0.758
*crf-1*	**−0.05 (0.57)** −1.26–1.07, *p* = 0.467	**0.12 (0.43)** −0.70–1.08, *p* = 0.611	**−0.31 (0.82)** −1.97–1.28, *p* = 0.350	**0.25 (0.47)** −0.64–1.22, *p* = 0.698
*crf-2*	**−0.87 (0.69)** −2.31–0.27, *p* = 0.088	**0.05 (0.44)** −0.88–1.00, *p* = 0.546	**1.16 (0.87)** −0.45–2.93, *p* = 0.918	**0.30 (0.47)** −0.57–1.29, *p* = 0.734
*crf-r1*	**0.04 (0.57)** −1.12–1.21, *p* = 0.520	**0.21 (0.44)** −0.58–1.20, *p* = 0.670	**0.39 (0.80)** −1.17–2.06, *p* = 0.686	**−0.37 (0.45)** −1.32–0.47, *p* = 0.204
*crf-r2*	**−0.11 (0.58)** −1.31–1.02, *p* = 0.420	**0.28 (0.46)** −0.53–1.34, *p* = 0.727	**0.10 (0.81)** −1.50–1.68, *p* = 0.546	**−0.31 (0.47)** −1.29–0.57, *p* = 0.260
*crh-bp*	**0.39 (0.61)** −0.72–1.72, *p* = 0.737	**0 (0.41)** −0.89–0.84, *p* = 0.494	**0.08 (0.80)** −1.49–1.67, *p* = 0.545	**−0.15 (0.46)** −1.10–0.72, *p* = 0.372
*ef*	**−0.13 (0.59)** −1.33–1.06, *p* = 0.414	**0.12 (0.43)** −0.68–1.09, *p* = 0.606	**0.38 (0.81)** −1.24–2.07, *p* = 0.688	**−0.1 (0.46)** −1.03–0.81, *p* = 0.411
*egr-1*	**−0.08 (0.59)** −1.31–1.07, *p* = 0.444	**−0.04 (0.43)** −0.95–0.84, *p* = 0.468	**−0.81 (0.85)** −2.55–0.76, *p* = 0.160	**0.16 (0.46)** −0.77–1.12, *p* = 0.632
*erk-1*	**−0.15 (0.57)** −1.34–0.97, *p* = 0.395	**−0.23 (0.44)** −1.25–0.52, *p* = 0.312	**0.14 (0.79)** −1.38–1.75, *p* = 0.567	**−0.22 (0.45)** −1.15–0.63, *p* = 0.318
*erk−2*	**0.10 (0.57)** −1.02–1.24, *p* = 0.570	**0.27 (0.47)** −0.55–1.36, *p* = 0.717	**0.65 (0.82)** −0.88–2.34, *p* = 0.788	**0.02 (0.45)** −0.90–0.92, *p* = 0.513
*gabaa*	**−0.07 (0.58)** −1.22–1.09, *p* = 0.454	**−0.08 (0.42)** −1.01–0.75, *p* = 0.426	**−1.62 (0.93)** −3.55–0.07, *p* = 0.035	**−0.36 (0.46)** −1.30–0.49, *p* = 0.214
*gapdh*	**0.09 (0.58)** −1.05–1.30, *p* = 0.564	**0.41 (0.50)** −0.38–1.56, *p* = 0.807	**0.53 (0.82)** −1.00–2.16, *p* = 0.745	**0.04 (0.47)** −0.93–0.99, *p* = 0.539
*gr1*	**−0.07 (0.59)** −1.33–1.04, *p* = 0.456	**−0.33 (0.46)** −1.37–0.44, *p* = 0.230	**−0.90 (0.85)** −2.59–0.68, *p* = 0.140	**−0.15 (0.45)** −1.06–0.75, *p* = 373
*gr2*	**0 (0.59)** −1.21–1.24, *p* = 0.490	**0.16 (0.44)** −0.66–1.12, *p* = 0.631	**0.30 (0.81)** −1.29–1.96, *p* = 0.649	**−0.24 (0.45)** −1.17–0.65, *p* = 0.299
*isopre*	**1.21 (0.79)** −0.07–2.87, *p* = 0.953	**0.12 (0.44)** −0.74–1.08, *p* = 0.599	**−0.79 (0.85)** 2.47–0.80, *p* = 0.170	**−0.02 (0.46)** −0.07–2.87, *p* = 0.953
*mr*	**−0.31 (0.61)** −1.64–0.89, *p* = 0.306	**−0.31 (0.47)** −1.39–0.45, *p* = 0.256	**0.20 (0.82)** −1.42–1.83, *p* = 0.603	**−0.25 (0.47)** −1.22–0.65, *p* = 0.298
*palld*	**0.11 (0.55)** −0.96–1.26, *p* = 0.577	**0.20 (0.45)** −0.61–1.22, 0.656	**−0.40 (0.83)** −2.08–1.18, *p* = 0.313	**−0.16 (0.46)** −1.10–0.76, *p* = 0.363
*pomc1*	**0.09 (0.60)** −1.08–1.38, *p* = 0.546	**−0.39 (0.50)** −1.58–0.39, *p* = 0.221	**1.47 (0.93)** −0.22–3.40, *p* = 952	**1.68 (0.73)** 0.28–3.13, *p* = 0.997
*pomc2*	**0.75 (0.71)** −0.41–2.31, *p* = 0.867	**−0.22 (0.44)** −1.23–0.56, *p* = 0.316	**2.05 (1.12)** 0.03–4.45, *p* = 0.977	**1.09 (0.63)** −0.01–2.40, *p* = 0.974
*prolr*	**−0.45 (0.61)** −1.78–0.66, *p* = 0.233	**0.14 (0.43)** −0.67–1.07, *p* = 0.618	**−0.99 (0.89)** −2.84–0.63, *p* = 0.118	**0.11 (0.46)** −0.77–1.07, *p* = 0.586
*serotr*	**−1.47 (0.87)** −3.27–0.02, *p* = 0.033	**−0.61 (0.58)** −1.96–0.21, *p* = 0.117	**−0.74 (0.85)** −2.44–0.87, *p* = 0.194	**−0.32 (0.47)** −1.30–0.54, *p* = 0.244

* = telencephalon, ** = hypothalamus, *** = optic tectum, **** = rhombencephalon.

## Data Availability

The datasets generated during and/or analysed during the current study are available from the corresponding author on reasonable request.

## Supplementary Materials

The following are available online at https://www.mdpi.com/2076-2615/11/2/585/s1, Figure S1: “Variable correlation plot of the PCA for the IEG-related genes in each in the 4 brain parts”, Figure S2: “Variable correlation plot of the PCA for the HPI axis-related genes in each in the 4 brain parts”, Figure S3: “Variable correlation plot of the PCA for the serotonin- and *gabaa*-related genes in each in the 4 brain parts”, Figure S4: “Posteriori probability plots of all genes in each in the 4 brain parts of control fish and fish 30 min after the air exposure”, Table S1: “Primer pairs selected for the gene expression studies”, Table S2: “Average expression stability (M value) of the potential reference genes”.
