# Supplementary material for: Distress Regulates Different Pathways in the Brain of Common Carp: A Preliminary Study
Source: Animals (Basel). 2021 Feb 23;11(2):585. doi: 10.3390/ani11020585 (PMC7926896; doi:10.3390/ani11020585)
Supplement: Supplementary file 1 [file animals-11-00585-s001.pdf]

Supplements

Table S1. Primer pairs selected for the gene expression studies.

Primer name	Sense Sequence (5′-3′) forward primer (f1)	Anti-Sense Sequence (5′-3) reverse primer (r1)
Reference genes		
eIF4E	GTCCTCCTTCGGTCACCATG	ATCATCCACTGCGCTCTTGT
ef a	AGTCCGTTGAGATGCACCAC	GGTCGTTCTTGCTGTCTCCA
bactin	AGACCTGTATGCCAACACCG	TTCATGGTGGAGGGAGCAAG
Immediate early genes		
c-fos	GACATCGCCAACCTGCTCAA	TTGCAGATGGGTTTGTGTGC
palld	CCAGAGACAGCGGTGATGAG	ATGCGGCAGAGTTTACCCTC
egr 1	CAGGGAAGATGTCTGGGCTG	TCATCCACATCCATCGCCAG
erk 1	TCGCTCCGTTTAGATGCTCG	GACAGGGCTATTGGATCGCA
erk 2	GCATCAACGACATCATCCGC	GGTCATTGCTCAAGTGCTGC
Metabolic gene		
gapdh	TCACAGCTACACAGAAGACC	GAGCTCAGGAATGACTTTGC
HPI axis-related genes		
crf-1	TACGCACAGATTCTCCTCGC	TCATACGGTGGTGGAAAGGC
crf-2	GCAACTAGAAGACAGACGCG	GCGAGGAGAATCTGTGCGTA
crf r1	GATGTTTGGAGAGGGCTGCT	CCAAGCGACGATAATGGGGA
crf r2	ATGAGACGAATGAGCCCTGG	CGTGTGAAGATAGCAGCCCT
crhbp	TTCACAGCAGAACGTCCACA	TGCAGTCGATGTTGACCGAA
pomc1	CGTCTACCCTGGAGAAAGCC	GGTCCATTTGCTCAGCAGGA
pomc2	CACCATCTGCCCTTCCACTT	AGTTGTGTTTGGAGCGGACT
gr1	AGCACAGTACCAAAGGACGG	TTTGCCCAGCTCCTTGATGT
gr2	TGCAGGACTTCGAGTTGGAC	GGCAAGTCTCCAAGCAAAGC
mr	CAGCCCTAACACCTGTAGCC	GAGGGCTGGAAACTGGAGAC
Serotonergic and gabanergic genes		
5-ht-r	AACCTCCAGCTCCACCTACT	CGAGCATGTTTCCATCGCTG
serotr	TGTCCCTGGCTACATCCTCA	CAGTCTGGTGAACTGCCTGA
gabaa	GGAGAGCGTGTAACCGAAGT	AGCTCTGCCTGAAGAACACA
isopre	CTCAAGTGGTGGTCTGCTGT	GTCGAGACTGCCAATCCTGT

NCBI accession numbers of the known sequences of carp (or closely-related fish species as indicated) that have been used for designing the primers using the Primer 3 software (Untergasser et al., 2012) are listed here: *eIF4E*: consensus build from two *C. carpio* eukaryotic translation initiation factor 4E-binding protein 2-like mRNA sequences (XM_019077307 and XM_019063850); *ef*: based on the known sequence of *C. carpio* (XM_019124726); *bactin*: based on the known sequences of *C. carpio* (JQ_619775 und JQ_619774); *c-fos*: based on the known sequence of *C. carpio* (U81505.2); *palld*: *C. carpio* cytoskeletal associated protein palladin, transcript variant X2 and its palladin-like (LOC109085690) mRNA (XM_019097544 and XM_019100227); *egr-1*: based on the *Danio rerio* early growth response 1 mRNA and the predicted *C. carpio* early growth response protein 1-like (LOC109046594), mRNA (NM_131248.1 and XM_019064348.1); *erk-1*: *C. carpio* cERK1 mRNA for extracellular signal regulated protein kinase 1, complete cds (AB006038.1); *erk-2*: *C. carpio* cERK2 mRNA for extracellular signal regulated protein kinase 2, complete cds (AB006039); gapdh: *C. carpio* partial mRNA for putative glyceraldehyde-3-phosphate dehydrogenase (AJ870982.1); *pomc1*: based on the *C. carpio* pro-opiomelanocortin-1 (LOC109076472) mRNA (XM_019092167.1), *pomc2*: based on the *C. carpio* pro-opiomelanocortin-2 (LOC109047265), transcript variants X1 and X2 (XM_019064966.1 and XM_019064968.1); *crf1*: *C. carpio* mRNA for putative corticotropin releasing hormone (AJ317955.1); *crf2: C. carpio* mRNA for putative corticotropin releasing hormone-2 (AJ576243.1); *crfr1*: *C. carpio* partial mRNA for putative corticotropin releasing hormone-receptor1 (crh-r1 gene) (AJ576244); *crfr2*: *C. carpio* partial mRNA for corticotropin releasing hormone-receptor 2 (crh-r2 gene) (AJ781795); *crh-bp*: mRNA for the putative corticotropin releasing hormone binding protein 1 in carp (AJ490880.1); *gr1*: *C. carpio* corticotropin releasing

factor receptor type 1.1 and type 1.2 mRNA, complete cds (FJ839679 and FJ839680); *gr2*: *C. carpio* partial mRNA for glucocorticoid receptor 2 (AM183668); *mr*: built on the predicted *C. carpio* mineralocorticoid receptor-like (LOC109105895) mRNA (XM_019119182.1); *prolr*: (); *5-ht-r*: based on the known 5-hydroxytryptamine receptor 2A-like sequence from *C. carpio* (XM_019063784), *gabaa*: based on the *C. carpio* gamma-aminobutyric acid receptor subunit alpha-1-like (LOC109046570), transcript variant X6 mRNA (XM_019064332.1); *isopre*: *C. carpio* isotocin precursor, gene, exons 1, 2 and 3 and complete cds (AF322651.2); *serotr*: derived from the *C. carpio* genome assembly common carp genome, scaffold: LG10, chromosome: 10 and the predicted sequence for the *C. carpio* sodium-dependent serotonin transporter-like (LOC109089953) mRNA (LN590700 and XM_019103831).

Table S2. Average expression stability (M value) of the potential reference genes summarized for all four brain regions (telencephalon, hypothalamus, optic tectum and rhombencephalon); the three genes with the lowest M value have been selected as reference genes.

Potential reference gene	geNORM M
beta actin	1.275
ef	1.310
eIF4E	1.480
egr-1	1.663
*b₂m**	1.735
palld	1.875
*18S RNA***	2.051
gapdh	2.338

**b₂m* primer pairs as published in Pietsch (2017)
***18S RNA* primer pairs (f1: CCGGGGCCATGATTAAGAGG; r1: AAGATCAGCCCGTTTGAGCA) built on the predicted *C. carpio* 18S mRNA partial sequence (NCBI accession number FJ710826)

References:

Pietsch, C. 2017. Zearalenone (ZEN) and its influence on regulation of gene expression in carp (*Cyprinus carpio* L.) liver tissue. Toxins 9, 283.

Untergasser, A., Cutcutache, I., Koressaar, T., Ye, J., Faircloth, B.C., Remm, M., Rozen, S.G. Primer3—New capabilities and interfaces. Nucleic Acids Res. 2012, 40, e115.

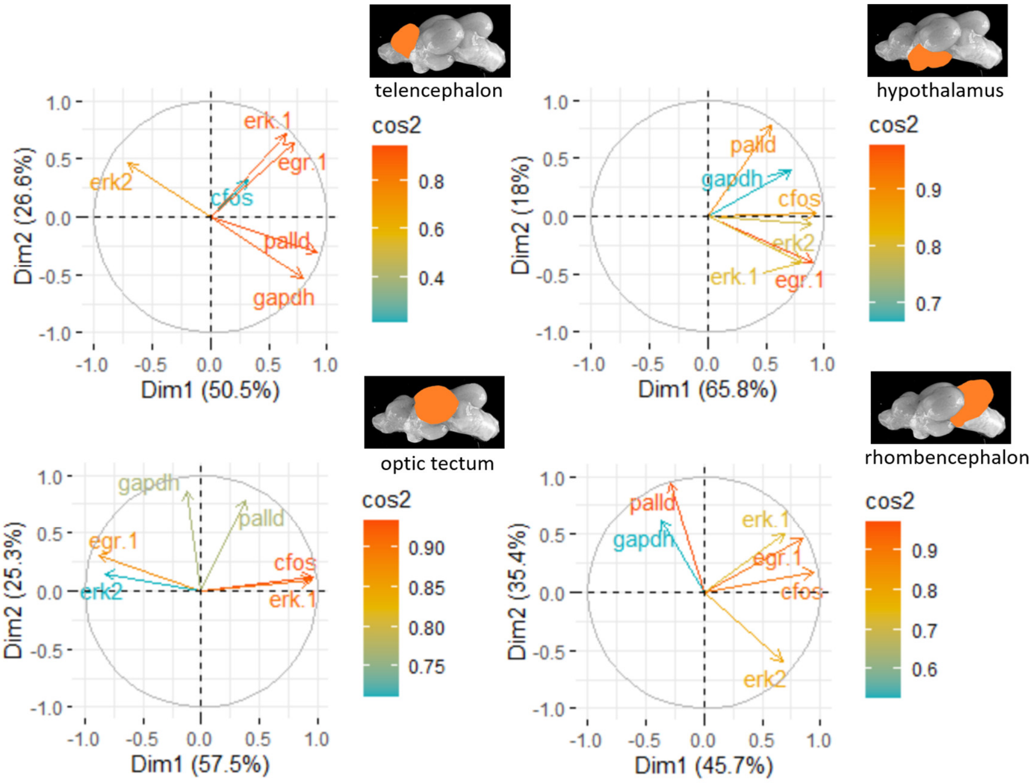

Figure Supplement S1. Variable correlation plot of the PCA for the IEG-related genes in each in the 4 brain regions of control fish and fish 30 min after the air exposure (the numbers in the brackets indicate the percentage of the variance in the data sets that is explained by the first two components Dim1 and Dim2) showing the quality of representation of the variables on the factor map cos^2, mean ± SEM; n = 4 per treatment.

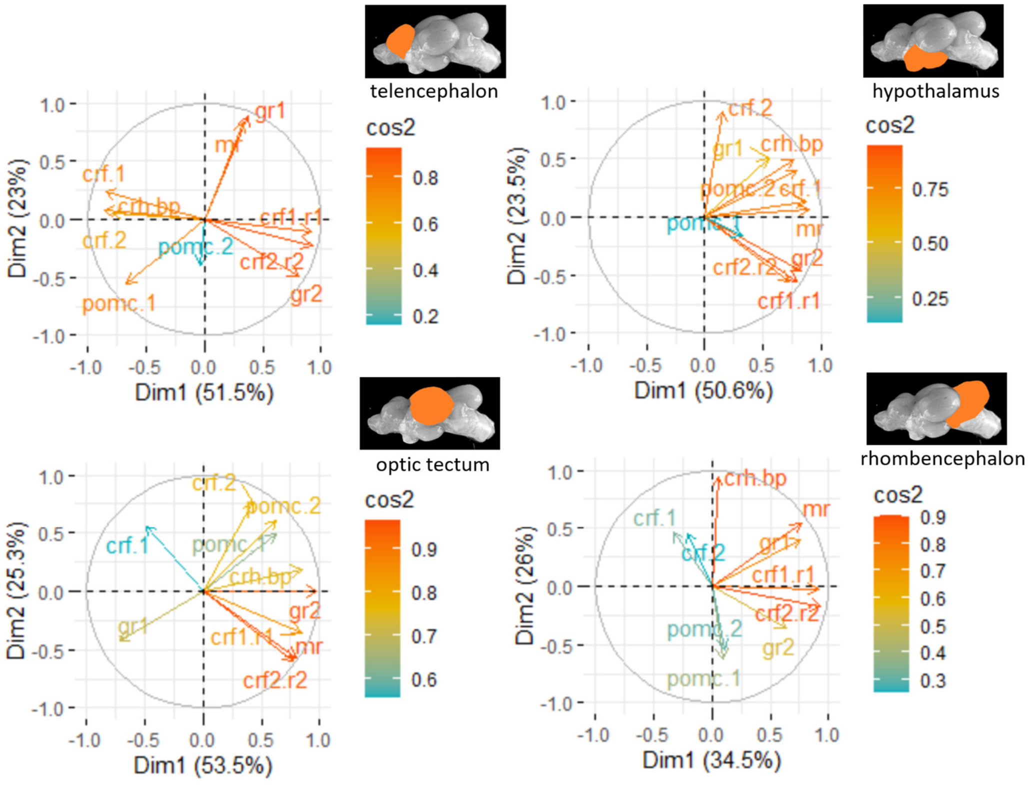

Figure Supplement S2. Variable correlation plot of the PCA for the HPI axis-related genes in each in the 4 brain regions of control fish and fish 30 min after the air exposure (the numbers in the brackets indicate the percentage of the variance in the data sets that is explained by the first two components Dim1 and Dim2) showing the quality of representation of the variables on the factor map cos^2, mean ± SEM; n = 4 per treatment.

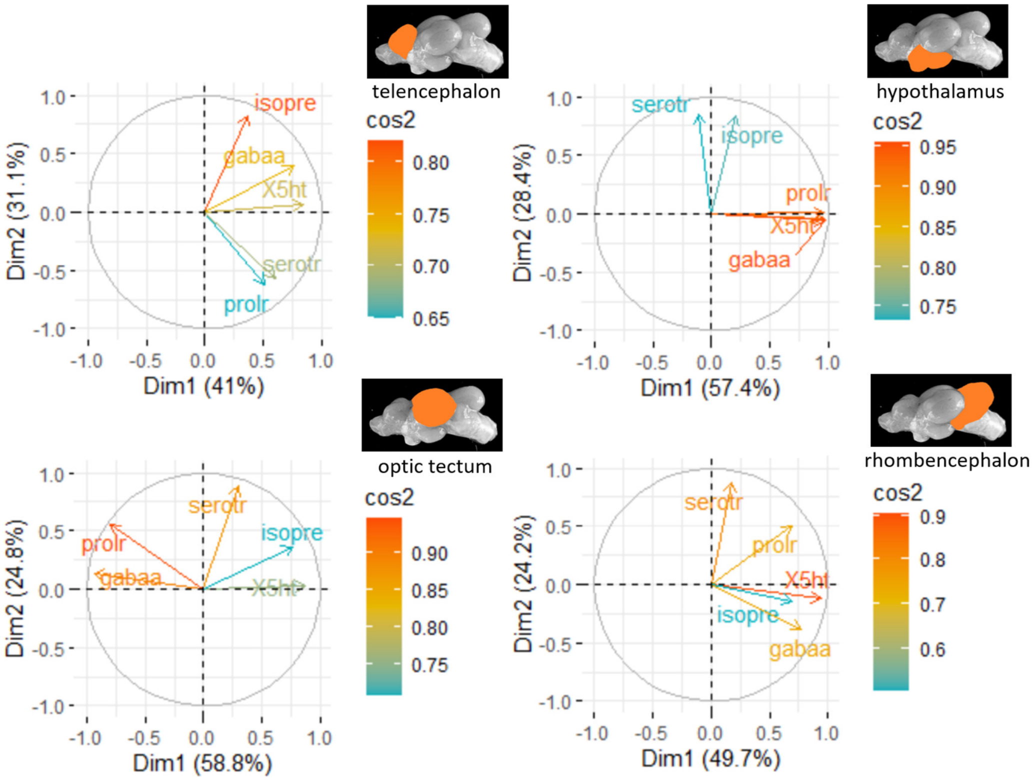

Figure Supplement S3. Variable correlation plot of the PCA for the serotonin- and *gabaa*-related genes in each in the 4 brain regions of control fish and fish 30 min after the air exposure (the numbers in the brackets indicate the percentage of the variance in the data sets that is explained by the first two components Dim1 and Dim2) showing the quality of representation of the variables on the factor map cos^2, mean ± SEM; n = 4 per treatment.

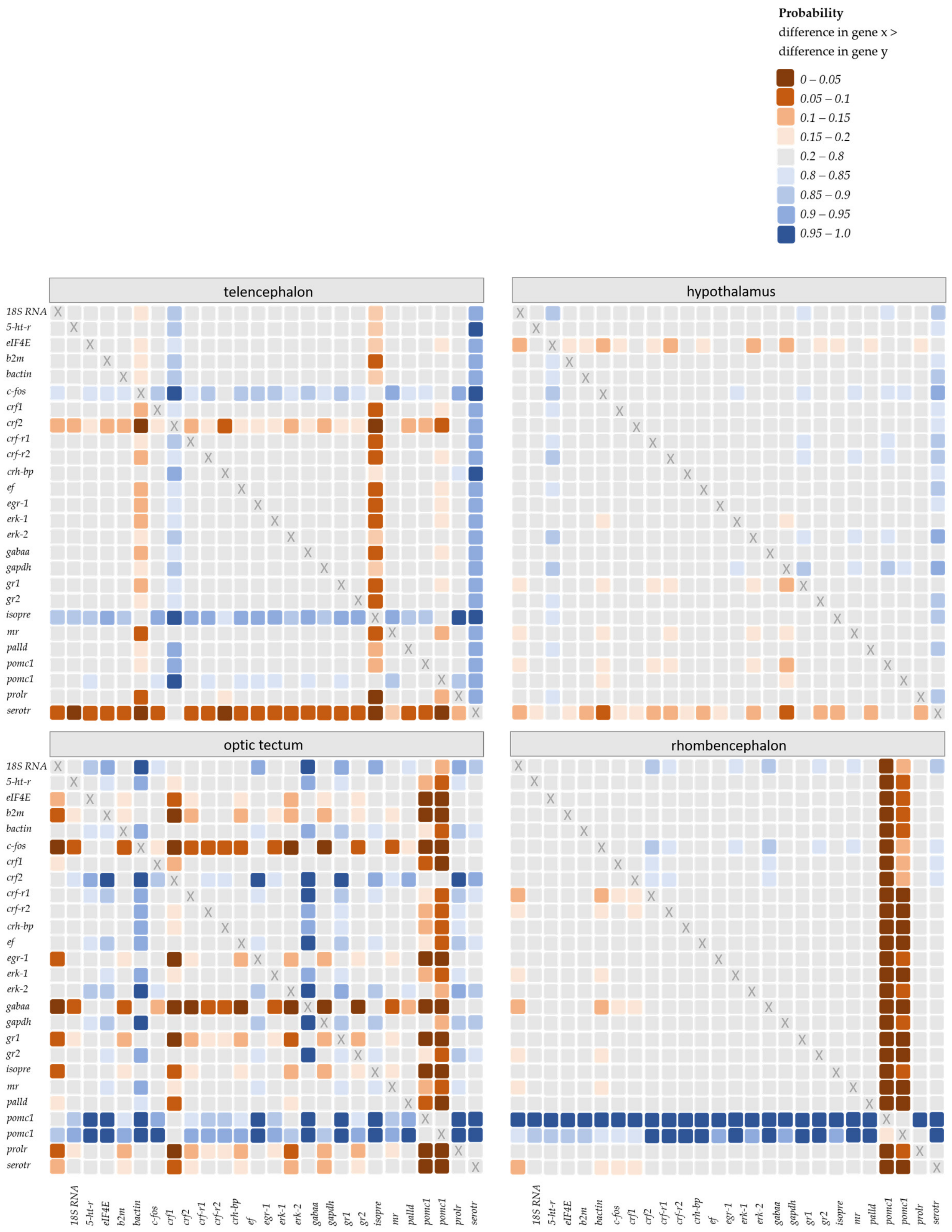

Figure Supplement S4. Posteriori probability plots of all genes in each in the 4 brain regions of control fish and fish 30 min after the air exposure representing the probability the gene x is showing higher values than gene y based on the Markov chain Monte Carlo (MCMC) realizations; n = 4 per treatment.
